# Extended Multicenter Study on the Postural Shirt for Women With Chronic Nonspecific Cervical Pain: A Randomized Crossover Clinical Trial

**DOI:** 10.7759/cureus.84629

**Published:** 2025-05-22

**Authors:** Merce Avellanet, Aurelia Mena, Esther Pages, Anna Boada-Pladellorens

**Affiliations:** 1 Physical Medicine and Rehabilitation, Hospital Nostra Senyora de Meritxell, Escaldes-Engordany, AND; 2 Physical Medicine and Rehabilitation, Complejo Hospitalario de Navarra, Pamplona, ESP

**Keywords:** cervical pain, crossover trial, exercise, neck pain, posture

## Abstract

Materials and methods

This is a multicenter randomized crossover study. This study included women between 21 and 55 years old with chronic nonspecific cervical pain (NCP) ≥3 on the visual analogue scale (VAS), able to wear the Medi Posture Plus Force (MPF) shirt, perform the exercises, and attend the follow-up assessments. Participants were allocated to either perform exercises (Ex group) or wear MPF (MPF group). The crossover between interventions was separated by a three-month washout period. We analyzed the effects of both interventions on pain intensity and posture as primary outcomes and neck disability (Neck Disability Index (NDI)), psychological factors (Pain Catastrophizing Scale (PCS)), and pain reliever intake as secondary outcomes.

Results

A total of 62 participants were randomized for sequencing, and after the two intervention periods, 56 were analyzed in the Ex group and 54 in the MPF group. Both interventions significantly improved pain (p = 0.001 for Ex and p < 0.010 for MPF). The mean NDI and PCS improved significantly in the Ex (p = 0.021 for NDI and p = 0.001 for PCS) and MPF groups (p = 0.042 for NDI and p < 0.010 for PCS). The intake of pain relievers was lower in the MPF group (8%) than in the Ex group (23.5%; p = 0.030).

Conclusion

MPF shirt was equal, and to some extent superior, to exercise for chronic NCP in women healthcare workers regarding pain intensity and other outcome measures. The use of MPF significantly decreased pain reliever intake compared to exercise.

## Introduction

According to the data from the Global Burden of Diseases, Injuries, and Risk Factors Study 2019, neck pain is the second most common skeletal-muscular disease [1]. Chronic nonspecific cervical pain (NCP) has been estimated to affect 45% to 54% of the general population sometime during their lives [1]. This disorder affects women more often than men, with a peak around middle age, causing a remarkable economic burden in terms of treatment costs, loss of productivity, and job-related issues [2]. Neck pain is one of the biggest contributors to musculoskeletal disability, impacting the physical, social, and psychological well-being of individuals [3]. In this sense, healthcare workers have been pointed out as a group especially inclined to suffer from chronic NCP [4].

Chronic NCP is defined as pain in and around the neck and shoulder girdle in the absence of infection, inflammatory, or structural pathology, such as fracture, that lasts for more than three months. The origin of chronic NCP is considered largely multifactorial and includes poor posture, anxiety, and depression [5,6]. Noteworthy, postural impairments in the thoracic spine can alter the mechanical loading of the cervical spine and cause neck problems, which is also highlighted by the increased incidence of neck issues in older adults with thoracic hyperkyphosis [7].

High-quality data regarding NCP management is scarce. A review of guidelines for treating neck pain revealed that exercise, cervical/thoracic manipulation and mobilization, and manual therapy are based on low-quality evidence [8]. A review covering clinical guidelines, conclusions of systematic reviews, and clinical expertise found that strategies including exercise and posture modification should be recommended to manage NCP [9,10].

Indeed, a recent meta-analysis on the effects of scapular treatment on chronic neck pain concludes that scapular therapy was beneficial for relieving pain intensity in patients with CNP, especially in women [11]. A German orthopedic company, Medi, launched Medi Posture Plus Force (MPF) based on the lessons learned from the spinal orthosis Spinomed, developed by the same company [12]. MPF is a T-shirt with tensional inelastic bands that aims to enhance abdominal and shoulder proprioception to improve posture. The MPF exercises slight traction for postural realignment, acting as a functional reminder, incentivizing contraction of abdominal and periscapular muscles based on an anatomical and physiological approach. Our group undertook a randomized crossover trial comparing MPF with exercise and determined that MPF had, at least, a similar effect on pain [13]. However, our conclusions were limited by the small sample size of the single-center study [14]. Therefore, we considered it appropriate to extend the study with a larger number of participants from another center to confirm or refute our previous findings.

In this extended study, we aimed to compare the MPF shirt against exercise, the standard of care for chronic NCP in women healthcare workers. We analyzed the effects of both interventions on pain intensity and posture as primary outcomes and neck disability and psychological factors as secondary outcomes. We also evaluated patients with dorsal hyperkyphosis separately and analyzed the impact of both interventions on pain reliever intake, compliance, and the effects of MPF shirt comfort.

## Materials and methods

Design

This was an extended study of a previously published work in a single center. Therefore, herein, we described a multicenter, randomized, crossover clinical trial undertaken at Hospital Nostra Senyora de Meritxell (HNSM), Andorra, and at Complejo Universitario de Navarra (CUN), Pamplona, between 2017 and 2022. The study was registered on clinicaltrials.gov (ID number: NCT03560492) and was approved by the Clinical Research Ethics Committees of HNSM and CUN, and a written informed consent was obtained from all participants.

Randomization and allocation concealment

A randomization plan was generated by a computer before the study started [14]. The assignment was performed by an independent assessor to guarantee allocation concealment and prevent selection bias. Participants were allocated at random to receive the following two interventions sequentially for three months: wear the MPF shirt (MPF group) or practice stretching and strengthening exercises involving cervical and dorsal areas (Ex group). A three-month washout period separated the crossover between the interventions (Ex or MPF groups). The random allocation sequence was generated before initiating the study, and participants were allocated consecutively without any restriction.

The design of the study, a crossover trial with interventions performed by participants, cannot be blinded intrinsically. To avoid investigator bias, several outcomes were patient-related outcome measures. For posture assessment, the investigator undertaking the Spinal Mouse® scan was blinded to the participant’s intervention group. We defined dorsal hyperkyphosis as a kyphotic angle of >45º. Further details can be found in the previously published study [13]. We reported the results of this study in agreement with the CONSORT statement for randomised crossover trials [15]. An external investigator included all the variables in an SPSS database, which was audited at three different times by two investigators and the statistician.

Participants, therapists, and centers

We recruited study participants through emails sent to nurses or allied health professionals from the two hospitals and associated health facilities. We evaluated the eligibility of the candidates during a complete medical consultation. We included women between 21 and 55 years old with chronic NCP (more than three months’ duration) rated ≥3 in the visual analogue scale (VAS) on average, able to wear the MPF garment, perform the exercises, and attend the follow-up assessments, who voluntarily signed informed consent. We excluded pregnant women; those with malignancies or other severe diseases, radiculopathy, spondylotic myelopathy, and psychiatric disorders; and those unable to perform the exercises or unwilling to comply with the follow-up.

Materials

The MPF is a T-shirt with tensional inelastic bands that exercise light traction on abdominal and periscapular muscles for postural realignment, focusing on an anatomical and physiological approach. The abdominal inelastic bands enhance core muscle contractions that facilitate a correct upright position and realign the cervical segment. Moreover, the dorsal bands are positioned in a functional anatomical direction following interscapular forces and enhancing perivertebral contractions. The breathable and highly elastic fabric comprises elastane, cotton, and polyamide [13].

Intervention

In the MPF group, we demonstrated to the participants how to wear the MPF garment and told them to wear it for two to four hours per day, during the daytime, every day of the week, under other clothing, for three months. The Ex group attended five sessions of 20 minutes (once a week for five weeks) with a physiotherapist to learn stretching and strengthening exercises of the cervical and dorsal areas. This group also received instructions to continue the exercises at home daily for three months. Physiotherapists from HNSM performed a training session for physiotherapists from CUN to ensure homogeneity in the exercises undertaken by participants at both hospitals. The exercise program included strengthening and stretching exercises.

After a three-month washout period, the participants swap (crossover) the intervention for another three months. We generated the random allocation sequence before initiating the study and allocated participants consecutively without any restriction [14]. Interventions in the extended study were undertaken between June 2021 and August 2022.

We controlled for possible cross-intervention contamination by reporting performed exercises and usual physical activity in the logbook provided at the inclusion. Furthermore, participants consented and agreed to follow the instructions for exercise or MPF during the study period.

Outcome measures

Primary Outcomes

Primary outcomes were pain intensity, measured with the VAS, and posture. The VAS is a validated measure of the construct of subjective pain intensity in patients with neck pain [16]. The temporal component of the pain intensity measured was the average pain felt during the previous seven days. The VAS used was a 100 mm horizontal line with verbal descriptors where participants graded the amount of pain from no pain (left 0 mm) to an extreme amount of pain (right 100 mm). We chose a 30% reduction or 20 mm reduction in neck pain measured with the numerical VAS as a clinically relevant change in pain intensity. We used a device for computerized measurement of surface curvature (Spinal Mouse®) in the upright position to objectively measure posture without the MPF shirt, which has proven its reliability and validity [17]. The investigator undertaking the Spinal Mouse® scan was blinded to the participant’s intervention group. We also analyzed separately those patients presenting dorsal hyperkyphosis (>45º), measured with the Spinal Mouse® scan.

Secondary Outcomes

Accurate pain assessment is critical to chronic pain conditions, including assessment of pain burden. Consequently, secondary outcomes included cervical pain-related disability, measured with the validated Spanish version of the Neck Disability Index (NDI) [18] and psychological factors, assessed with the Pain Catastrophizing Scale (PCS) [19]. We assessed the global perceived effect of treatment (exercise and MPF shirt) with a VAS, a 100 mm horizontal line with verbal descriptors where participants graded the amount of perceived effect of treatment from “no effect” (left 0 mm) to “maximum improvement” (right 100 mm). We evaluated the MPF shirt comfort with a five-point Likert-type scale questionnaire ranging from very uncomfortable to very comfortable. We assessed primary and secondary outcomes at one, 30, 60, and 90 days after the intervention. After the washout period, we also measured the outcomes at the same intervals for the following intervention. The same investigators recorded the outcomes before and after the crossover. We provided all participants with a logbook to record treatment compliance, medication intake (number and type of analgesic medication for NCP), adverse events, and other comments. Compliance was measured as a percentage according to intervention adherence. We considered that a participant was compliant with the intervention when she performed the exercises or wore the shirt at least 75% of the time we asked her to.

Data analysis

We expressed continuous variables as the mean and standard deviation (SD) and discrete variables as absolute frequencies and percentages. The required sample size to achieve a two-point difference or 30% reduction in the VAS for pain with an SD of 2.5 was calculated to be 30 participants in the previous study. In this extended study, the objective was to increase the sample size. We analyzed between-treatment differences for each patient within each sequence and across both sequences. To estimate the treatment effect, we employed mixed models for repeated measurements. The model incorporated time, treatment arm, and the interaction between treatment and time as fixed effects. Patient factors were introduced as a random effect in the intercept to account for individual variability. The correlation structure of the variance-covariance matrix was specified as unstructured. We used the Student’s t-test and linear regression for quantitative variables with a normal distribution and the Welch’s test for quantitative variables with a non-normal distribution. For categorical variables, we used the chi-squared test and, for continuous variables, Fisher’s exact test. The statistical significance level was set at p < 0.05. An independent researcher performed the data analyses, which a second statistician reviewed to ensure proper execution and compliance with the planned analysis. Missing data were treated as missing values.

## Results

Flow of participants, therapists, and centers through the study

Patient Characteristics at Baseline and After Washout

**Figure 1:**
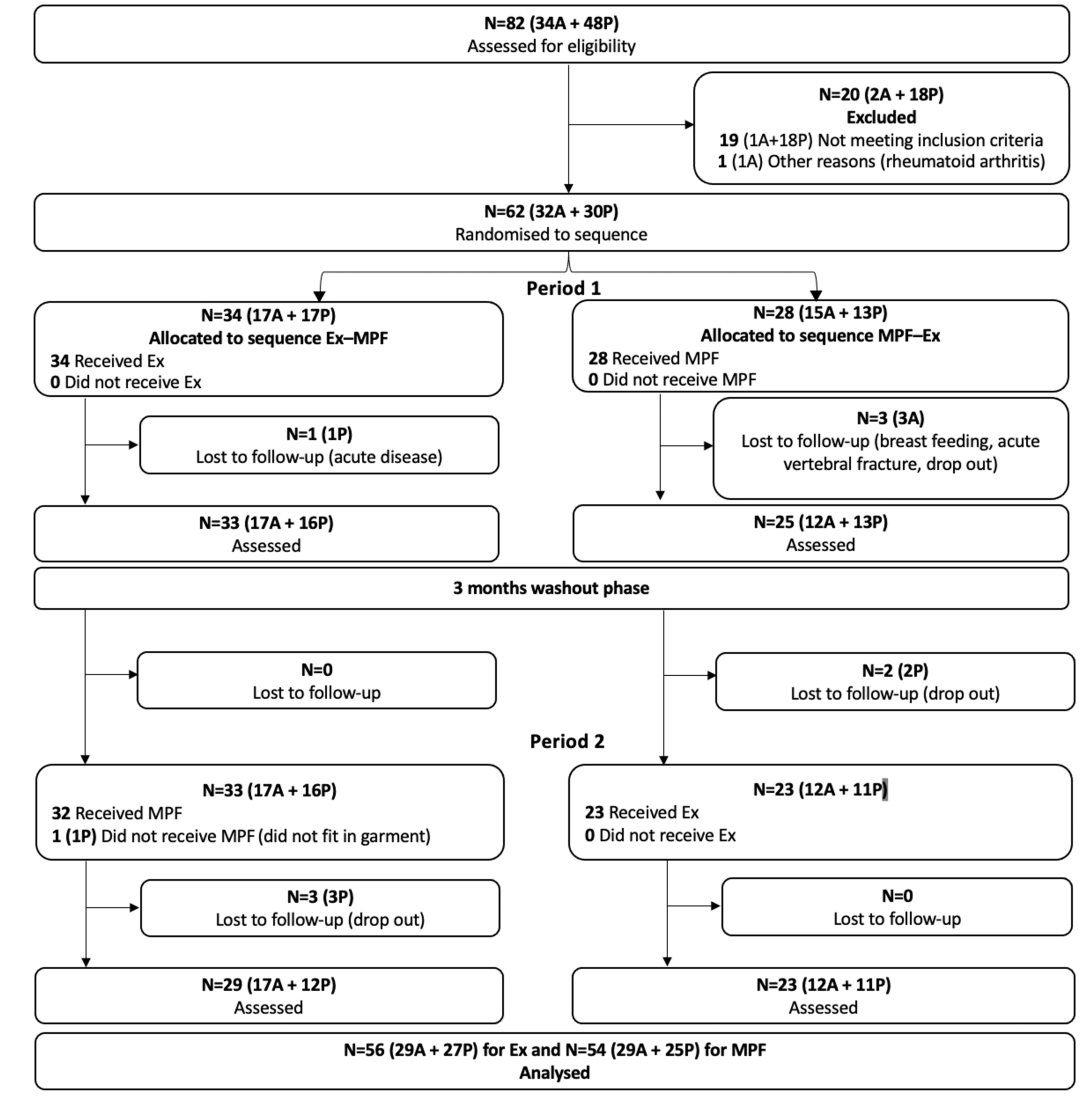
Patient enrolment flowchart A: patients recruited at the Hospital Nostra Senyora de Meritxell (Andorra); Ex: exercise; MPF: Medi Posture Plus Force garment; P: patients recruited at the Complejo Universitario de Navarra (Pamplona)

A total of 82 participants were assessed for eligibility, and 62 were randomized to sequence. After the two intervention periods, 56 participants were analyzed in the Ex group and 54 in the MPF group (Figure 1). No differences in the studied variables were detected between the MPF and Ex groups at baseline or after the washout period, except for a higher body mass index in the Ex group at baseline that was also present after the washout in the MPF group (Table 1). Changes within participants after the end of intervention 1 were reversed after the end of the washout period (Table 2).

**Table 1:** Demographic and clinical characteristics of study participants at baseline and after the three-month washout period BMI: body mass index; Ex: exercise; MPF: Medi Posture Plus Force garment; PCS: Pain Catastrophizing Scale; SD: Standard deviation Figures are mean (and SD). Statistically significant (<0.05) p-values are indicated in bold. The p-values were calculated with the Welch t-test

	Baseline			After washout beginning period 2		
	Ex (N = 33)	MPF (N = 25)	p-value	Ex (N = 23)	MPF (N = 29)	p-value
Age (years)	43.6 (9.1)	42.0 (8.7)	0.508	41.6 (8.9)	43.3 (9.1)	0.496
BMI (kg/m^2^)	26.50 (6.56)	22.82 (2.22)	0.010	22.57 (2.12)	26.00 (6.04)	0.012
Visual analogue scale	5.48 (1.98)	5.25 (1.65)	0.643	4.83 (2.02)	4.46 (2.33)	0.540
Dorsal spine Spinal mouse (degrees)	51.09 (9.90)	50.04 (12.1)	0.736	44.48 (10.27)	48.39 (11.31)	0.198
Lumbar spine Spinal mouse (degrees)	-33.61 (11.73)	-32.96 (14.56)	0.852	-31.09 (13.03)	-35.55 (13.43)	0.227
Inclination Spinal mouse (degrees)	-0.58 (4.58)	0.72 (5.60)	0.338	0.74 (3.92)	0.52 (3.88)	0.836
Neck Disability Index	12.73 (4.57)	10.96 (4.78)	0.158	10.13 (4.65)	10.63 (6.07)	0.745
PCS	14.70 (9.99)	10.60 (8.90)	0.111	5.65 (6.03)	8.38 (7.50)	0.157

**Table 2:** Clinical characteristics of study participants at the end of treatment period 1 and after the three-month washout period MPF: Medi Posture Plus Force garment; SD: standard deviation Figures are mean (and SD). Statistically significant (<0.05) p-values are indicated in bold. The p-values were calculated with the Welch t-test

	Exercise (N = 33)			MPF (N = 25)		
	End period 1	After washout	p-value	End 1^st^ treatment	After washout	p-value
Visual analogue scale	3.72 (2.57)	4.46 (2.33)	0.228	2.74 (1.86)	4.83 (2.02)	<0.001
Neck Disability Index	9.64 (6.03)	10.63 (6.07)	0.513	7.88 (4.74)	10.13 (4.65)	0.107
Pain Catastrophizing Scale	6.42 (6.86)	8.38 (7.5)	0.278	3.88 (4.62)	5.65 (6.03)	0.257
Dorsal spine Spinal mouse (degrees)	47.3 (9.42)	48.39 (11.31)	0.678	45.12 (12.37)	44.48 (10.27)	0.847
Lumbar spine Spinal mouse (degrees)	-34.61 (14.33)	-35.55 (13.43)	0.787	-35.84 (17.70)	-32.09 (13.03)	0.298
Inclination Spinal mouse (degrees)	-1.30 (4.08)	0.52 (3.88)	0.073	0.36 (3.5)	0.74 (3.92)	0.725

Primary Outcomes: Pain Intensity and Posture

No adverse events were reported for either of the two interventions. According to the VAS, the mean pain intensity improved significantly in the Ex group (at baseline: 5.22, SD: 2.00; after exercise: 3.72, SD: 2.60; p = 0.001) and in the MPF group (at baseline: 4.81, SD: 2.08; after MPF: 3.14, SD: 2.04; p < 0.010) (Table 3). The mean dorsal and lumbar spine and the inclination measured with the Spinal Mouse® did not improve in either the Ex or the MPF group (Table 3).

**Table 3:** Pain intensity, neck disability, psychological status, and posture of study participants before and after interventions AI: after intervention; Base.: baseline; MPF: Medi Posture Plus Force garment; PCS: Pain Catastrophizing Scale; SD: standard deviation Figures are mean (and SD) unless otherwise stated. Statistically significant (<0.05) p-values are indicated in bold. The p-values were calculated with the Welch t-test

	Exercise			MPF			
	Base. (N = 56)	AI (N = 56)	p-value	Base. (N = 54)	AI (N = 54)		p-value
Visual analogue scale	5.22 (2.00)	3.72 (2.60)	0.001	4.81 (2.08)	3.14 (2.04)		<0.010
% Pain improvement	N/A	25.5	N/A	N/A	26.5		N/A
Dorsal spine Spinal mouse (degrees)	51.09 (9.90)	47.70 (10.05)	0.095	47.27 (14.79)	48.11 (11.33)		0.739
Lumbar spine Spinal mouse (degrees)	-32.57 (12.32)	-34.23 (12.06)	0.471	-34.39 (13.88)	-34.81 (14.22)		0.875
Inclination Spinal mouse (degrees)	-0.04 (4.33)	-0.98 (4.12)	0.239	0.61 (4.69)	-0.65 (3.53)		0.117
Neck Disability Index	11.66 (4.74)	9.05 (6.80)	0.021	10.77 (5.50)	8.68 (5.15)		0.042
PCS	10.98 (9.60)	5.68 (7.04)	0.001	9.35 (8.16)	4.54 (4.67)		<0.010

Secondary Outcomes: Neck disability and Psychological Status

After the intervention, the mean NDI improved significantly in the Ex (p = 0.021) and the MPF groups (p = 0.042). Likewise, the mean PCS improved notably in the Ex (p = 0.001) and the MPF groups (p < 0.01) (Table 3).

Dorsal Hyperkyphosis, Pain Relievers, Compliance, and Comfort

Regarding participants with dorsal hyperkyphosis, the pain was lower in the Ex group (p = 0.038) and in the MPF group (p = 0.003) after the intervention. Pain improvement was higher in the MPF group (56.5%) than in the Ex group (43.5%). The spinal surface curvature did not improve in either of the two groups. The NDI did not improve significantly in either of the two groups, but the PCS was significantly lower in the Ex (p = 0.014) and the MPF groups (p = 0.001) (Table 4).

**Table 4:** Pain intensity, disability, psychological status, and posture of participants with dorsal hyperkyphosis (>45º) before and after interventions AI: after intervention; MPF: Medi Posture Plus Force garment; PCS: Pain Catastrophizing Scale; SD: standard deviation Figures are mean (and SDs) unless otherwise stated. Statistically significant (<0.05) p-values are indicated in bold. The p-values were calculated with the Welch t-test

	Exercise			MPF		
	Base. (N = 35)	AI (N = 32)	p-value	Base. (N = 34)	AI (N = 31)	p-value
Visual analogue scale	5.15 (2.12)	3.93 (2.58)	0.038	4.77 (1.89)	3.28 (2.03)	0.003
% Pain improvement	N/A	43.5	N/A	N/A	56.5	N/A
Dorsal spine Spinal mouse (degrees)	54.57 (7.42)	53.97 (6.86)	0.732	56.38 (8.29)	55.52 (8.41)	0.678
Lumbar spine Spinal mouse (degrees)	-35.09 (10.44)	-36.66 (12.15)	0.572	-37.18 (11.05)	-37.16 (10.53)	0.996
Inclination Spinal mouse (degrees)	-0.66 (4.43)	-1.59 (4.37)	0.385	-0.26 (4.93)	-1.65 (3.40)	0.198
Neck Disability Index	11.51 (4.84)	9.28 (6.20)	0.104	11.41 (5.77)	8.80 (5.56)	0.071
PCS	11.89 (10.50)	6.28 (7.11)	0.014	10 (7.75)	4.23 (4.65)	0.001

Remarkably, the percentage of patients taking more pain relievers than before the intervention was higher in the Ex (73.7%) with respect to the MPF group (26.3%; p = 0.026) (Table 5).

**Table 5:** Variation on the intake of pain relievers relative to baseline MPF: Medi Posture Plus Force garment Figures are absolute numbers (and %). The p-value was calculated with the chi-squared test

	Exercise (N = 56)	MPF (N = 54)	p-value	Total (N = 110)
More	14 (73.7)	5 (26.3)	0.026	19 (17.3)
Less or no change	42 (46.2)	49 (53.8)		91 (82.7)

**Figure 2:**
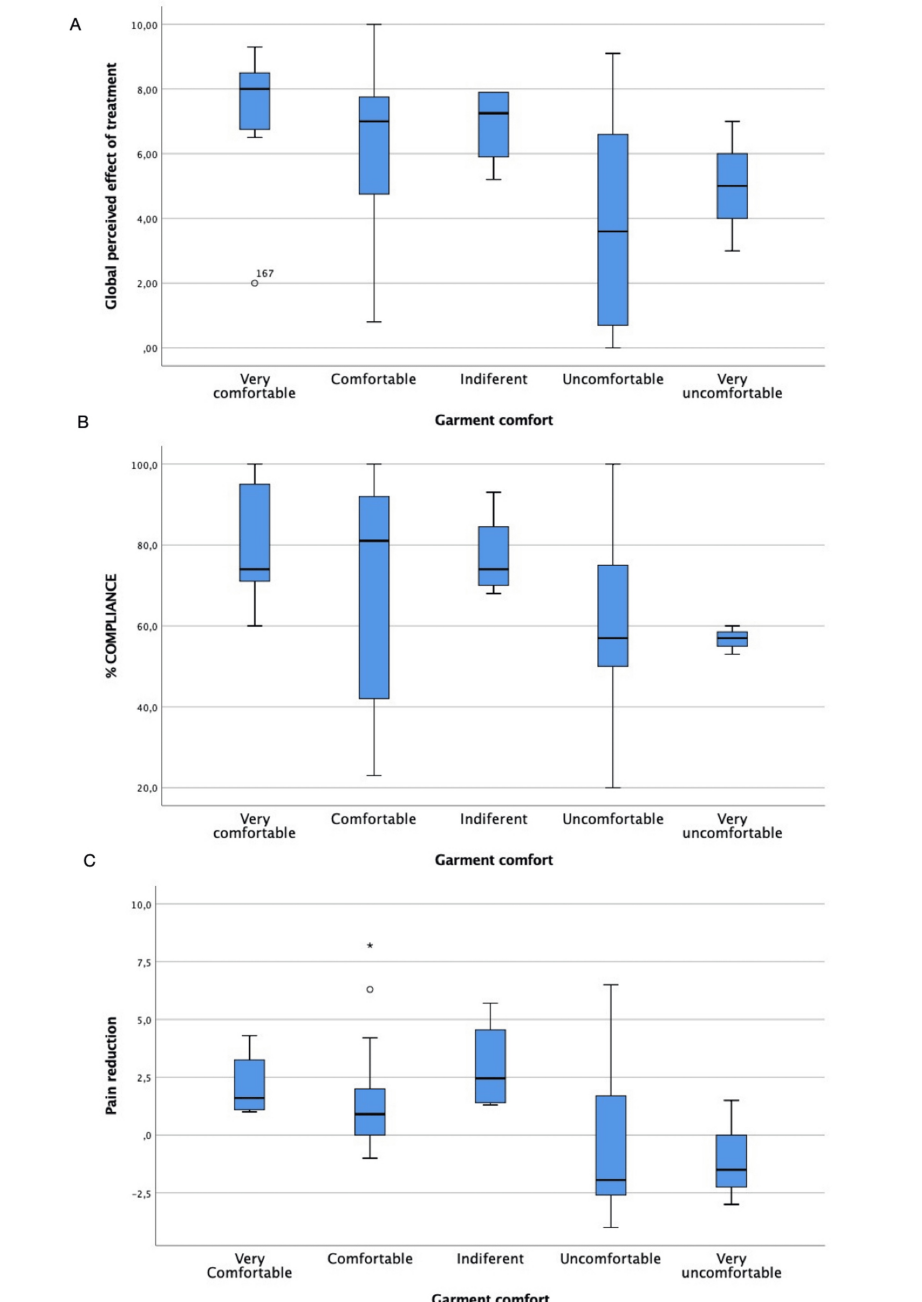
Box-and-whisker plot of (A) the global perceived effect of treatment (assessed with a visual analogue scale), (B) compliance, and (C) pain reduction (assessed with a visual analogue scale) according to Medi Posture Plus Force shirt comfort

Regarding the perceived comfort of the MPF shirt, a tendency was observed with higher values of global perceived effect of treatment (p < 0.010), compliance (p = 0.038), and pain reduction (p = 0.016) in those participants who found the shirt more comfortable (Figure 2).

Compliance with the trial method

Compliance with the intervention (wearing the garment at least two hours per day or executing the daily exercises) was determined when participants appropriately followed the prescribed indications for >75% of the planned time. A total of 36 (64%) participants complied with the intervention in the Ex group and 37 (69%) in the MPF group.

Follow-up after the end of the study

In a non-preplanned analysis, 56 participants were asked whether they continued wearing the MPF shirt and/or doing the exercises four months after the end of the study, and 37 (66%) answered the question. A total of 14 (37.8%) participants did not continue either of the two interventions; 12 (32.5%) continued wearing the MPF shirt; six (16.2%) continued both, i.e., wearing the shirt and doing the exercises; and five (13.5%) kept performing only the exercises.

## Discussion

In this extended study, we evaluated the MPF shirt against exercise, the standard of care for chronic NCP in female healthcare workers. Both the MPF shirt and exercise improved pain intensity to a similar extent. Besides, both interventions had a comparable positive impact on neck disability and psychological status. Although there was no improvement in the spinal posture, pain improved more in participants with dorsal hyperkyphosis. Interestingly, the intake of pain relievers was lower in the MPF group than in the Ex group. Therefore, the two interventions could not be considered equivalent since participants wearing the MPF shirt had a similar improvement in pain to those performing exercises but needed fewer pain relievers.

Pain improvement

As previously reported, the MPF shirt was safe and showed a reduction in pain intensity comparable to that of exercise [13]. In this extended study, the mean percentage of pain improvement was lower (25.5% for Ex and 26.5% for MPF) than that of the previous study (33%) with fewer participants [13]. In fact, the aim of the extension of the study with a larger number of participants was to confirm or refute our previous findings. Although both the MPF shirt and exercise improved pain intensity to a similar extent, which confirms the findings, a possible explanation for the lower mean percentage difference may be due to the period of time when the extended study took place. That is during the COVID-19 pandemic period, and participants were health workers in a stressful situation. However, the similarity of these percentages between both groups implied that MPF was not inferior to exercise regarding pain improvement.

Posture

When analyzing the results of the crossover study, the mean dorsal and lumbar spine and the inclination measured with the Spinal Mouse® did not improve in either the Ex or the MPF group. However, during the first three-month period of the study, the MPF group improved the dorsal spine measure (50.04ª to 45.12º) more than the Ex group (51.09º to 47.30º), although this difference was not statistically significant. Moreover, after the washout, participants who wore the MPF in the first period maintained the slight improvement at the beginning of the second intervention (Table 1). In this sense, although MPF did not seem to induce any structural changes in the patients, it did improve their posture. The rationale of our study, as shown in the literature, is that posture improvement can have a positive effect on treating chronic NCP [20,21].

Unpublished data on biomechanical analysis of MPF use in 10 sedentary professional computer users (“Ferrari PR. Pilot study on the evaluation of the effects induced by the use of the postural shirt “Posture + shirt”) demonstrated that the postural behavior correction is due to muscular contraction. As already mentioned, scapular exercises are beneficial for relieving pain intensity in patients with CNP, especially in women [11].

In our study, posture did not change significantly with MPF use but improved pain. We hypothesize that MPF tensional inelastic bands seem to enhance periscapular muscular contraction. Consequently, MPF might act as a functional reminder, promoting proprioception and contraction periscapular muscles, and this leads to pain improvement.

Other orthotic devices improving neck pain have been previously reported, but they were either added to an exercise program, were studied for a very short period, and were chosen for a specific task (smartphone use or laptop use, or required a visit to a certified physiotherapist [22-25]. Nonetheless, all these devices showed promising results in their settings. Two similar studies with postural garments have been undertaken. In the first, a lumbosacral support showed a significant improvement in neck pain, but this randomized controlled trial lacked the crossover design usually considered highly efficient in rehabilitation research [26]. In the second study, deep cervical flexor muscle endurance was reported with a posture support jacket, but these results were achieved only in healthy participants [27].

Secondary outcomes

As shown in our previous study, both MPF and exercise significantly improved the disability and pain catastrophizing of the participants [13]. Studies evaluating the effects of posture supports have usually not included the analysis of the NDI. The lack of use of this questionnaire in studies evaluating orthotic devices was striking since it is commonly used in clinical trials to measure the functional status of patients with neck pain. Only one study assessing the effect of Tasuki (a sort of sash used to hold up the sleeves of traditional Japanese clothes) on neck pain found that the participants’ NDI improved significantly after intervention [23].

In relation to participants with dorsal hyperkyphosis, no differences were found between both arms, improving pain and PCS but with no effect on spinal surface curvature and NDI (Table 4). Noteworthy, in this subgroup, the percentage of pain improvement is higher than in the entire group, being more than double for the MPF arm (56% improvement vs 26.5%).

Regarding the intake of pain relievers, our results confirmed the previous findings that pointed to a significantly greater effect of MPF than exercise [13]. Analgesic medication intake in our population was on demand, specifically for cervical pain, as recorded in the logbook. The registration of this medication was done on a daily basis. The improvement in catastrophizing and perceived disability is observed in both groups, but the reduction in medication is only in the MPF group.

Compliance and perceived effect of treatment

The compliance in both groups of our study was very high, taking into account that we only considered those who followed the prescribed indications at least 75% of the time to be compliant. The participants benefiting more from the MPF shirt were those who found it comfortable (increasing their compliance and pain reduction), which resulted in a better global effect of treatment. Consequently, those who considered the shirt comfortable (83.3% of the sample) benefited most from its use. The other 16.6% found the shirt uncomfortable or very uncomfortable and experienced fewer benefits of its use.

Interestingly, our study, which included more participants than the original, confirmed, after the end of the study, that women were more compliant with the MPF shirt than with exercise in a non-preplanned analysis. Engaging in physical activity and exercise can help alleviate chronic pain [11,28]. Nevertheless, women tend to be less active than men and are often less consistent with exercise routines [2,28]. In our research, we found that women were more likely to stick to wearing the MPF shirt than to following an exercise regimen. This suggests that those who are reluctant to perform the stretching and strengthening exercises could benefit the most from wearing the shirt.

Despite the benefits observed with both interventions for chronic pain, 38% of the participants who answered a follow-up survey after the end of the study returned to being completely inactive and did not continue either of the two interventions. In contrast, of those continuing with one intervention, more than 50% choose voluntarily to wear MPF to improve NCP, and 22% continued to do only exercises. They reported finding MPF to be less time-consuming compared to exercising. 

Limitations

The results of our study should also be interpreted considering its limitations. The main limitation stemmed from the crossover design of the study, i.e., the possibility of order effects (which intervention the participant was receiving first) and carryover effects (the possibility of residual effects), although the latter was not very likely with a three-month washout period. To the best of our knowledge, there is no evidence on the washout period needed between exercise interventions [29]. Interestingly, in a recent crossover trial with different intensities of exercise for post-COVID-19 condition, the washout period was two to four weeks between intensive exercise sessions [30]. However, this has not been included as a limitation of the study, nor the blinding. Although cross-intervention contamination was controlled via the logbook, and the participants agreed to follow the instructions, it could not be completely excluded. In crossover designs, the emphasis should be on within-group changes rather than between-group differences.

Another relevant limitation is the sample size calculation. The approach to expected effect size was challenging given that there were no previous well-designed studies on the garment, and we couldn’t find, to the best of our knowledge, the effect size of exercise on cervical pain. We chose the crossover study design to increase statistical power, following statistician recommendations.

In addition, the dorsal hyperkyphosis measure is structural, and it could be that taking this measure with the MPF shirt on could have shown an improvement in posture. Besides, whole-day posture would certainly be different from a single isolated measure.

Posture supports, such as MPF, seem to represent a valuable alternative for populations with a high incidence of neck pain (e.g., women healthcare workers). However, considering that the MPF shirt did not fit into one participant, orthotic garments are limited compared to exercise and would be inferior to it when targeting a large population unless these garments were produced tailored instead of a one-size-fits-all.

## Conclusions

The MPF shirt was equal, and to some extent superior, to exercise to manage chronic NCP pain in women healthcare workers regarding pain intensity, posture, neck disability, and psychological status, but was better concerning pain reliever intake. According to our results, those who would benefit the most from wearing the MPF shirt are those who find the garment comfortable. However, size fitting could be a problem when studied in larger populations.
